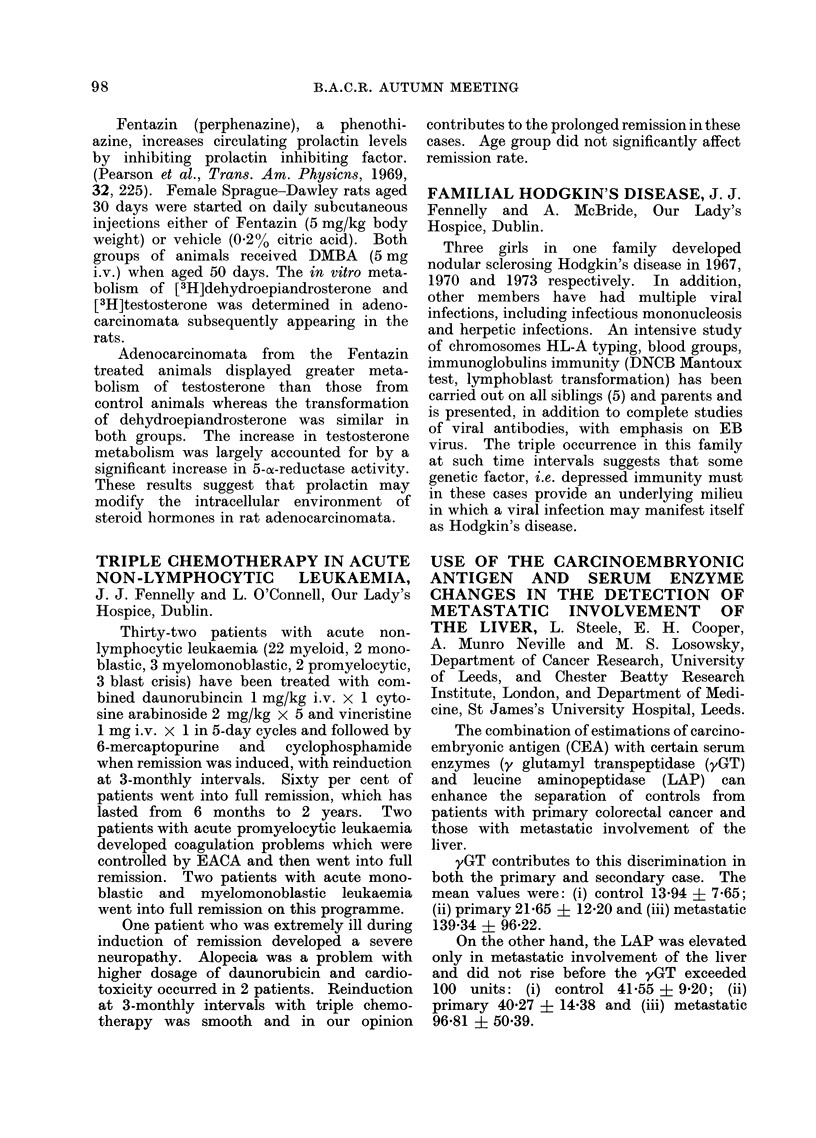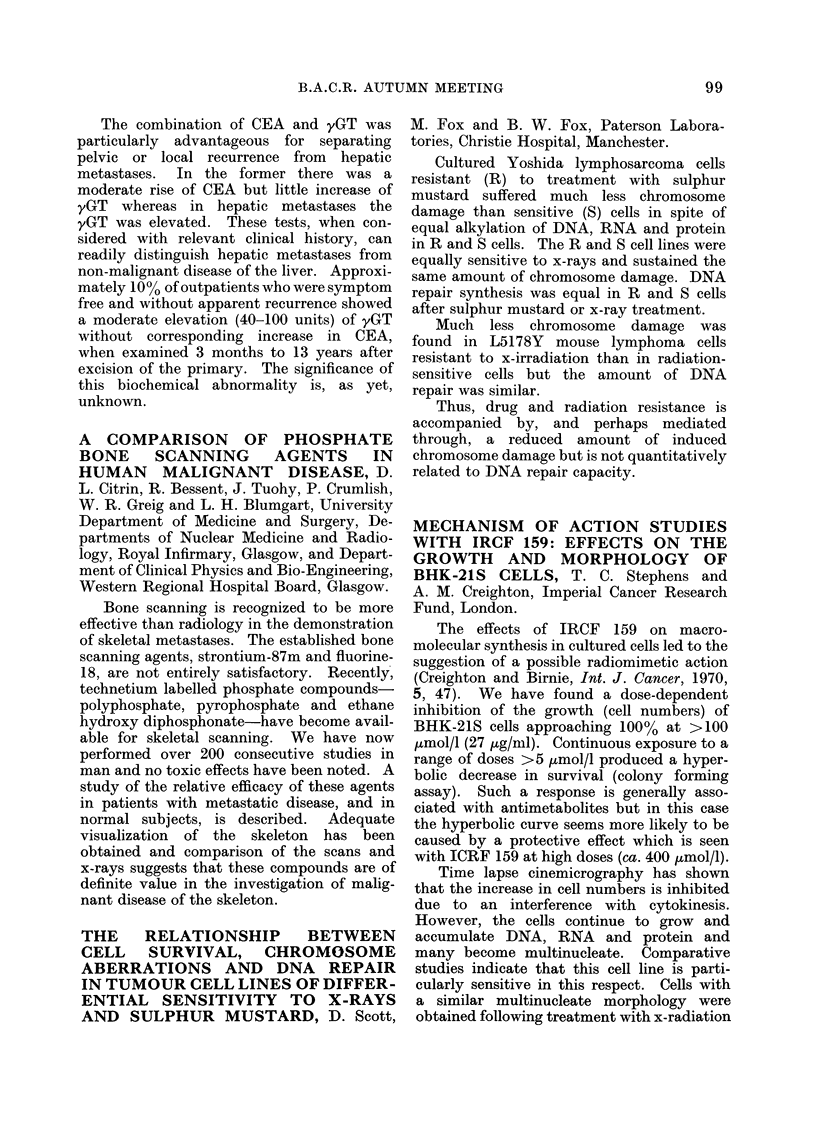# Proceedings: Use of the carcinoembryonic antigen and serum enzyme changes in the detection of metastatic involvement of the liver.

**DOI:** 10.1038/bjc.1974.36

**Published:** 1974-01

**Authors:** L. Steele, E. H. Cooper, A. M. Neville, M. S. Losowsky


**USE OF THE CARCINOEMBRYONIC ANTIGEN AND SERUM ENZYME CHANGES IN THE DETECTION OF METASTATIC INVOLVEMENT OF THE LIVER,** L. Steele, E. H. Cooper, A. Munro Neville and M. S. Losowsky, Department of Cancer Research, University of Leeds, and Chester Beatty Research Institute, London, and Department of Medicine, St James's University Hospital, Leeds.

The combination of estimations of carcinoembryonic antigen (CEA) with certain serum enzymes ($\gamma$ glutamyl transpeptidase ($\gamma$GT) and leucine aminopeptidase (LAP) can enhance the separation of controls from patients with primary colorectal cancer and those with metastatic involvement of the liver.

$\gamma$GT contributes to this discrimination in both the primary and secondary case. The mean values were: (i) control 13·94 ± 7·65; (ii) primary 21·65 ± 12·20 and (iii) metastatic 139·34 ± 96·22.

On the other hand, the LAP was elevated only in metastatic involvement of the liver and did not rise before the $\gamma$GT exceeded 100 units: (i) control 41·55 ± 9·20; (ii) primary 40·27 ± 14·38 and (iii) metastatic 96·81 ± 50·39.

The combination of CEA and γGT was particularly advantageous for separating pelvic or local recurrence from hepatic metastases. In the former there was a moderate rise of CEA but little increase of γGT whereas in hepatic metastases the γGT was elevated. These tests, when considered with relevant clinical history, can readily distinguish hepatic metastases from non-malignant disease of the liver. Approximately 10% of outpatients who were symptom free and without apparent recurrence showed a moderate elevation (40–100 units) of γGT without corresponding increase in CEA, when examined 3 months to 13 years after excision of the primary. The significance of this biochemical abnormality is, as yet, unknown.